# Protective Effect of Sulfated Polysaccharides from Celluclast-Assisted Extract of *Hizikia fusiforme* Against Ultraviolet B-Induced Skin Damage by Regulating NF-κB, AP-1, and MAPKs Signaling Pathways In Vitro in Human Dermal Fibroblasts

**DOI:** 10.3390/md16070239

**Published:** 2018-07-17

**Authors:** Lei Wang, WonWoo Lee, Jae Young Oh, Yong Ri Cui, BoMi Ryu, You-Jin Jeon

**Affiliations:** 1 Department of Marine Life Sciences, Jeju National University, Jeju 63243, Korea; comeonleiwang@163.com (L.W.); ojy0724@naver.com (J.Y.O.); chyr6019@126.com (Y.R.C.); 2 Freshwater Bioresources Utilization Division, Nakdonggang National Institute of Biological Resources, Sangju 37242, Korea; 21cow@naver.com

**Keywords:** *Hizikia fusiforme*, sulfated polysaccharides, ultraviolet-B, MMPs, NF-κB, AP-1, MAPKs

## Abstract

Our previous study evaluated the antioxidant activities of sulfated polysaccharides from Celluclast-assisted extract of *Hizikia fusiforme* (HFPS) *in vitro* in Vero cells and *in vivo* in zebrafish. The results showed that HFPS possesses strong antioxidant activity and suggested the potential photo-protective activities of HFPS. Hence, in the present study, we investigated the protective effects of HFPS against ultraviolet (UV) B-induced skin damage *in vitro* in human dermal fibroblasts (HDF cells). The results indicate that HFPS significantly reduced intracellular reactive oxygen species (ROS) level and improved the viability of UVB-irradiated HDF cells in a dose-dependent manner. Furthermore, HFPS significantly inhibited intracellular collagenase and elastase activities, remarkably protected collagen synthesis, and reduced matrix metalloproteinases (MMPs) expression by regulating nuclear factor kappa B (NF-κB), activator protein 1 (AP-1), and mitogen-activated protein kinases (MAPKs) signaling pathways in UVB-irradiated HDF cells. These results suggest that HFPS possesses strong UV protective effect, and can be a potential ingredient in the pharmaceutical and cosmetic industries.

## 1. Introduction

In humans, skin is the largest organ of the integumentary system. It undergoes chronological aging similar to other organs. Skin is in direct exposure to the outside environment and therefore it undergoes aging as a consequence of environmental damage [1]. Ultraviolet (UV) irradiation from sunlight is the primary environmental factor that induces human skin aging and results in pigment accumulation and wrinkle formation. Human skin is frequently affected by oxidative stress caused by continuous exposure to UV irradiation from sunlight. Skin exposed to UV and environmental oxidizing pollutants is associated with diverse abnormal reactions including inflammatory responses, epidermal hyperplasia, the breakdown of collagen, and melanin accumulation [2,3].

UV can be classified into three subtypes of UVA, UVB, and UVC, based on the wavelength. UVB has a medium wavelength and is thought to bring more cellular stress to humans compared to the other two subtypes [4,5,6]. UVB is known to be associated with human health through stimulating reactive oxygen species (ROS) generation [7,8]. The excessive ROS subsequently activate cell signaling pathways including nuclear factor kappa B (NF-κB), activator protein 1 (AP-1), and mitogen-activated protein kinases (MAPKs), which stimulate matrix metalloproteinases (MMPs) expression [9]. MMPs are a class of structurally similar enzymes, and play a major role in physiological and pathological tissue remodeling. The imbalance of MMP expression could lead to cartilage, cardiac, and cancer-related diseases [10]. MMPs degrade the collagenous extra cellular matrix (ECM) in connective tissues, which is the main factor of wrinkling. Therefore, an ideal MMP inhibitor or an agent that reduces the expression of MMPs may be effective against wrinkle formation, and could be thought as a promising candidate to be used as an ingredient in the cosmetic industry.

Marine organisms are rich resources of several natural compounds such as polyphenol, polysaccharide, sterol, and peptide, which possess various bioactivities including antioxidant, anticancer, anti-inflammatory, anti-obesity, antihypertensive, anti-diabetes, and UV protective activities [11,12,13,14,15,16,17,18,19,20]. *Hizikia fusiforme* (*H. fusiforme*) is an edible brown seaweed, which is distributed in the areas of the northwest Pacific, including Korea, China, and Japan. *H. fusiforme* has been utilized as a traditional medicine and functional food. It contains various compounds, which possesses several of bioactivities, especially polysaccharides. Many studies have reported that polysaccharides from *H. fusiforme* possess various bioactivities such as antioxidant, anti-inflammatory, anti-angiogenic, anticancer, osteoprotective, and immunostimulatory activities [21,22,23,24]. Our previous research displayed that sulfated polysaccharides from Celluclast-assisted extract of *H. fusiforme* (HFPS) possess strong free radical scavenging activity and protective effects on H_2_O_2_-induced oxidative stress *in vitro* in Vero cells and *in vivo* in zebrafish [25]. These results suggest that HFPS may possess photo-protective activity. However, the protective effects of HFPS against UVB-induced skin damage have not yet been reported. Thus, the purpose of the present study was to investigate the protective effect of HFPS against UVB-induced skin damage *in vitro* in human dermal fibroblasts (HDF cells).

## 2. Results and Discussion

### 2.1. HFPS Inhibits Collagenase from Clostridium Histolyticum and Elastase from Porcine Pancreas

Both collagenase and elastase belong to proteases that break down proteins. Collagenase is the enzyme that breaks the peptide bonds in collagen, which is the key component of animal ECM. Elastase is the enzyme that breaks down elastin, which is an elastic fiber. Collagen and elastin together determine the mechanical properties of the connective tissue. In human skin, degradation of collagen leads to decreased skin thickness, and degradation of elastin results in losing skin elasticity. These are the major characteristics of wrinkle formation in aged skin. Thus, a collagenase or elastase inhibitor could be thought as an agent to reduce skin aging.

**Figure 1:**
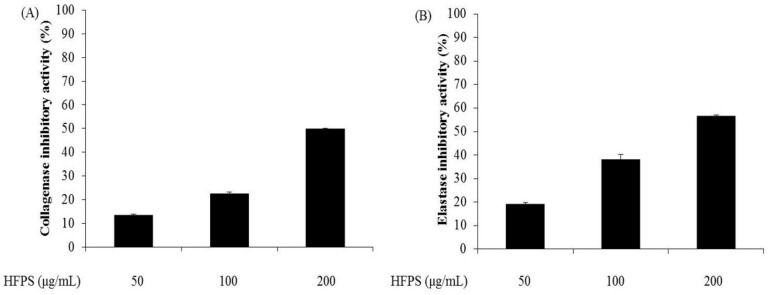
HFPS inhibits commercial collagenase and elastase: (**A**) collagenase inhibitory activity of HFPS; and (**B**) elastase inhibitory activity of HFPS. The experiments were conducted in triplicate, and the data are expressed as the means ± standard error (SE).

In this study, the inhibitory effects of HFPS against commercial collagenase and elastase were measured. As shown in Figure 1, HFPS inhibits collagenase and elastase in a dose-dependent manner. The collagenase inhibitory rates of HFPS were 13.46%, 22.58%, and 49.77% at the concentrations of 50, 100, and 200 μg/mL, respectively (Figure 1A); and the elastase inhibitory rates of HFPS were 18.78%, 38.14%, and 56.53% at the concentrations of 50, 100, and 200 μg/mL, respectively (Figure 1B). These results indicate that HFPS may possess the activity against skin aging through inhibition of collagenase and elastase.

### 2.2. HFPS Promotes HDF Cell Proliferation and UVB Irradiation Damages HDF Cells

Skin is continuously exposed to UVB from sunlight, thus it is a major target of oxidative stress [26]. UVB-induced skin cell oxidative stress leads to cell damage, resulting in skin photoaging. Over the past decades, with industrial development, the oxidizing pollutants in the air and UV irradiated to the earth have increased. This is becoming a serious issue that threatens the skin health of humans. Therefore, finding nontoxic and effective agents that can protect dermic damage would be valuable for medical and cosmetic industries.

**Figure 2:**
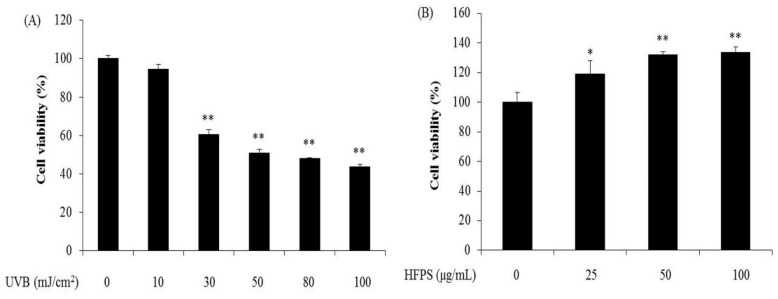
UVB irradiation damages HDF cells and HFPS promotes HDF cell proliferation: (**A**) cytotoxicity of UVB irradiation on HDF cells; and (**B**) proliferation effect of HFPS on HDF cells. Cell viability was measured by 3-(4-5-dimethyl-2yl)-2-5-diphenyltetrazolium bromide (MTT) assay. The data are expressed as the means ± SE (*n* = 3), * *p* < 0.05, ** *p* < 0.01 as compared to control group.

As the first step to evaluate protective effects and mechanisms of HFPS against UVB-induced skin damage in HDF cells, we employed various levels of UVB irradiation to induce cell damage in HDF cells, and determined the cytotoxicity of HFPS at different concentrations on HDF cells. As Figure 2 shows, UVB irradiation significantly decreased the viability of HDF cells in a dose-dependent manner (Figure 2A). Furthermore, the optimal UVB dose applied to HDF cells based on the 50% growth inhibitory dose was determined to be 50 mJ/cm^2^. We then assessed the effect of HFPS alone on HDF cells. The cytotoxicity results (Figure 2B) suggest that HFPS is non-toxic on HDF cells and promotes HDF cells proliferation in a dose-dependent manner. This promotion of proliferation was comparable in cells treated with 100 μg/mL and 50 μg/mL samples. From these results, 50 mJ/cm^2^ was determined as the optimal UVB dose applied to HDF cells and 100 μg/mL was selected as the maximum concentration of HFPS for further study.

### 2.3. HFPS Improves Cell Viability and Scavenges Intracellular ROS Generated in UVB-Irradiated HDF Cells

UVB irradiation induces skin damage though stimulated intracellular ROS generation [27]. Recently, identification of ROS scavengers from natural resources has been given more attention. Our previous study isolated phlorotannins from marine algal, *Ecklonia cava*, and evaluated their UV protective effect. The results displayed that phlorotannins reduced intracellular ROS induced by UVB irradiation and improved cell viability in a dose-dependent manner [26]. Zeng et al. investigated the protective effect of polysaccharides from *Ganoderma lucidum* against UVB-induced photoaging. The results suggest that polysaccharides from *Ganoderma lucidum* significantly reduced ROS levels and improved the viability of UVB-irradiated cells [28].

**Figure 3:**
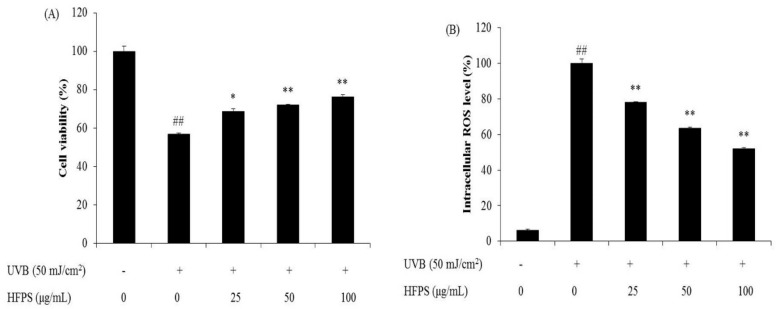
Protective effects of HFPS against UVB-induced HDF cell damage: (**A**) protective effects of HFPS against UVB-induced HDF cell damage; and (**B**) intracellular ROS scavenging effect of HFPS in UVB-irradiated HDF cells. Cell viability was measured by MTT assay and intracellular ROS level was measured by DCF-DA assay. The data are expressed as the means ± SE (*n* = 3). * *p* < 0.05, ** *p* < 0.01 as compared to UVB-exposed group and ^##^ *p* < 0.01 as compared to control group.

In the present study, HDF cells were pretreated with HFPS and irradiated with UVB at a dose of 50 mJ/cm^2^. Cell viability was subsequently measured by MTT assay and intracellular ROS level was analyzed by DCF-DA assay. As Figure 3 shows, cell viability was decreased while intracellular ROS level was increased after UVB irradiation. Treatment with increasing concentrations of HFPS (25, 50, and 100 μg/mL) improved cell viability by 11.78%, 14.97%, and 19.21% (Figure 3A) and intracellular ROS scavenging by 21.95%, 36.44%, and 48.14% (Figure 3B). These results indicate that HFPS possesses a protective effect against UVB-induced cellular damage via ROS clearance in HDF cells.

### 2.4. HFPS Inhibits Intracellular Collagenase and Elastase Activities in UVB-Irradiated HDF Cells

**Figure 4:**
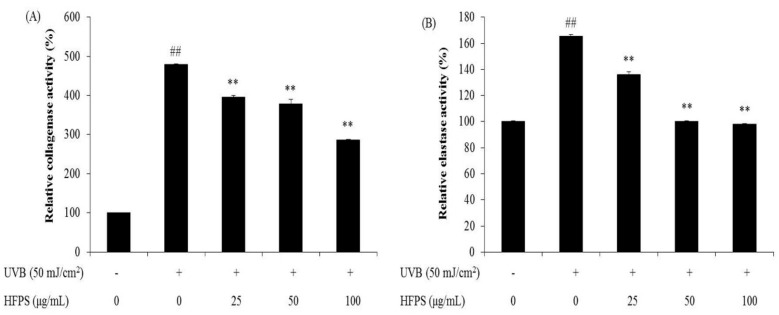
HFPS inhibits intracellular collagenase and elastase activities in UVB-irradiated HDF Cells: (**A**) relative collagenase activity; and (**B**) relative elastase activity. The data are expressed as the means ± SE (*n* = 3). * *p* < 0.05, ** *p* < 0.01 as compared to UVB-exposed group and ^##^ *p* < 0.01 as compared to control group.

Aged skin is thin and inelastic due to degradation of collagen and elastin in the ECM of connective tissue. Collagenase and elastase are key enzymes during collagen and elastin degradation. UVB stimulates the activities of fibroblast collagenase and elastase in the dermis consequently causing wrinkle formation [29,30]. As Figure 4 shows, the relative collagenase and elastase activities of UVB-irradiated HDF cells were significantly increased compared with non-irradiated cells. However, relative activities of both enzymes were decreased in the cells pretreated with HFPS in a dose-dependent manner. These results suggest that HFPS may act as an inhibitor of fibroblast collagenase and elastase and may prevent wrinkle formation induced by UVB irradiation.

### 2.5. HFPS Protects Collagen Synthesis and Reduces MMPs Expression Levels in UVB-Irradiated HDF Cells

MMPs, particularly MMP-2 and MMP-9, have been identified as being central to degradation of ECM [31]. In addition, MMP-1 degrades two major structural proteins, type I and type III collagen [32]. Collagen is synthesized as a precursor molecule, procollagen, which contains additional peptide sequences. These sequences are cleaved off during collagen secretion, thus, a number of sequences can indirectly reflect collagen synthesis level. We determined procollagen type I carboxy-terminal peptide (PIP) to investigate collagen synthesis level.

**Figure 5:**
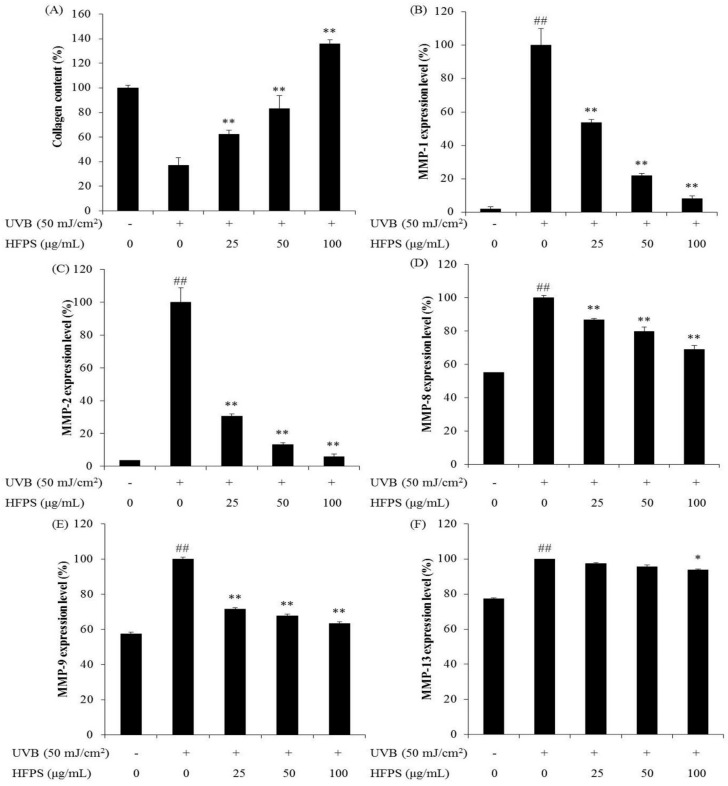
HFPS improve collagen synthesis and reduces MMPs expression in UVB-irradiated HDF cells: (**A**) collagen synthesis level in UVB-irradiated HDF cells; (**B**) MMP-1 expression level in UVB-irradiated HDF cells; (**C**) MMP-2 expression level in UVB-irradiated HDF cells; (**D**) MMP-8 expression level in UVB-irradiated HDF cells; (**E**) MMP-9 expression level in UVB-irradiated HDF cells; and (**F**) MMP-13 expression level in UVB-irradiated HDF cells. Collagen synthesis level was reflected by the amounts of PIP, and the amounts of PIP and MMPs were measured by the commercially ELISA kits, based on the manufacturer’s instructions. The data are expressed as the means ± SE (*n* = 3). * *p* < 0.05, ** *p* < 0.01 as compared to UVB-exposed group and ^##^ *p* < 0.01 as compared to control group.

As Figure 5 shows, UVB irradiation significantly decreased collagen synthesis in HDF cells, and HFPS dose-dependently protects collagen synthesis (Figure 5A). Furthermore, MMPs’ expression levels were significantly increased in UVB-irradiated HDF cells but decreased in HFPS pretreated cells (Figure 5B–F). These results suggest that HFPS effectively protects collagen synthesis and reduces the expression of MMPs.

### 2.6. HFPS Inhibits Nuclear Factor Kappa B (NF-κB) Activation, Reduces Activator Protein 1 (AP-1) Phosphorylation, and Suppresses Mitogen-Activated Protein Kinases (MAPKs) Activation in UVB-Induced HDF Cells

**Figure 6:**
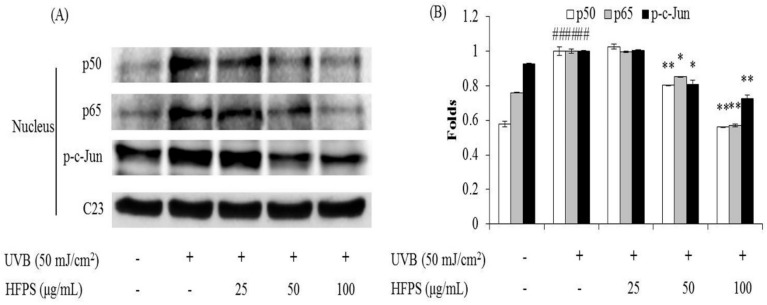
HFPS inhibits UVB-induced NF-κB activation and reduces AP-1 phosphorylation in UVB-irradiated HDF cells: (**A**) the inhibitory effects of HFPS on UVB-induced NF-κB related (p65 and p50) protein expression and AP-1 phosphorylation; and (**B**) relative amounts of NF-κB expressions and AP-1 phosphorylation levels. The relative amounts of NF-κB expressions and AP-1 phosphorylation were compared with C23. The data are expressed as the means ± SE (*n* = 3). * *p* < 0.05, ** *p* < 0.01 as compared to UVB-exposed group and ^##^ *p* < 0.01 as compared to control group.

NF-κB is a protein complex that controls cytokine production, transcription of DNA, and cell survival. NF-κB plays an important role in immune responses, and dysregulation of NF-κB is associated with various diseases such as cancer, inflammation, and aging [33]. Many studies have reported that UVB irradiation can activate NF-κB, and the activation of NF-κB can induce MMPs expression [34,35,36]. As Figure 6 shows, UVB irradiation significantly increases nuclear levels of NF-κB (p65 and p50); however, HFPS treatment remarkably reduces nuclear NF-κB levels in UVB-irradiated HDF cells in a dose-dependent manner.

**Figure 7:**
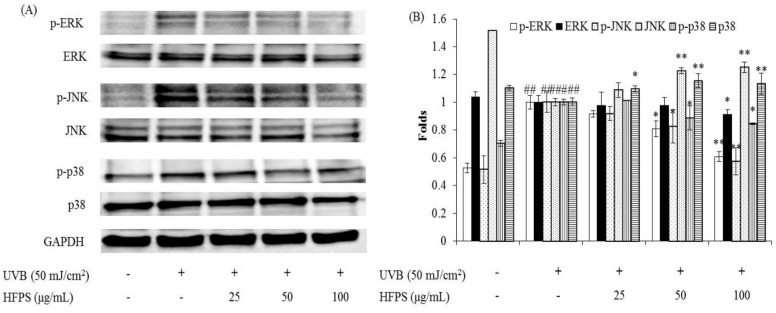
HFPS suppress MAPKs activation in UVB-irradiated HDF cells: (**A**) the inhibitory effects of HFPS on UVB-induced MAPKs activation; and (**B**) relative amounts of activated MAPKs levels. The relative amounts of activated MAPKs levels were compared with GAPDH. The data are expressed as the means ± S.E (*n* = 3). * *p* < 0.05, ** *p* < 0.01 as compared to UVB-exposed group and ^##^ *p* < 0.01 as compared to control group.

MAPKs are a type of protein kinases, which are involved in directing cellular responses to different stimuli, such as heat shock, mitogens, and pro-inflammatory cytokines. MAPKs regulate various cell functions including proliferation, differentiation, gene expression, cell survival and apoptosis [37,38]. The activation of MAPKs occurs through the phosphorylation of p38, Jun N-terminal kinase (JNK), and extracellular-regulated protein (ERK) signaling pathways. AP-1(c-Jun) is a nuclear transcription factor, which is phosphorylated after the activated MAPKs were translocated to the nucleus [39]. Subsequently, the expression of MMPs is up-regulated [33,40]. In the present study, the activated AP-1 and MAPKs levels were detected by Western blot analysis. The results indicate that UVB irradiation significantly phosphorylates AP-1 and HFPS remarkably reduces the phosphorylated AP-1 (p-c-Jun) levels in a dose-dependent manner (Figure 6). In addition, HFPS treatment effectively suppresses UVB-induced p38, JNK, and ERK phosphorylation in UVB-irradiated HDF cells (Figure 7). These results indicate that HFPS regulates NF-κB activation, AP-1 phosphorylation, and MAPKs activation in UVB-induced HDF cells.

## 3. Materials and Methods

### 3.1. Materials and Reagents

The fluorescent probe 2’, 7’-dichlorodihydroflurescin diacetate (DCFH-DA), dimethyl sulfoxide (DMSO), 3-(4-5-dimethyl-2yl)-2-5-diphynyltetrasolium bromide (MTT), 1 × phosphate buffered saline (PBS), collagenase from clostridium histolyticum, elastase from porcine pancreas, azo dye-impregnted collagen, and N-succinyl-Ala-Ala-Ala-p-nitroanilide were purchased from Sigma Co. (St. Louis, MO, USA). The Dulbecco’s modified Eagle medium (DMEM), Ham’s Nutrient Mixtures medium (F-12), penicillin/streptomycin, and fetal bovine serum (FBS) were purchased from Gibco BRL (Life Technologies, Burlington, ON, Canada). Antibodies against GAPDH, C23, p-c-Jun, NF-κB p65 and NF-κB p50, ERK and phospho-ERK, JNK and phospho-JNK, and p38 and phospho-p38 were purchased from Santa Cruz Biotechnology (Santa Cruz, CA, USA). Anti-rabbit IgG antibodies was purchased from Cell Signaling Technology (Beverly, MA, USA). PIP ELISA kit was purchased from TaKaRa Bio Inc. (Kusatsu, Japan) and Human MMP-1, 2, 8, 9, and 13 ELISA kits were purchased from GE Healthcare Life Sciences (Exeter*,* Devon, UK). All other chemicals used in this study were of analytical grade.

HFPS were prepared in our previous study. The separation and analysis procedures were described by Wang et al. [25]. In brief, the lyophilized *H. fusiforme* was hydrolyzed by Celluclast (Sigma, St. Louis, MO, USA, ≥700 units/g) at the optimal condition (pH 4.5, 50 °C) for 24 h and the polysaccharides (HFPS) were obtained by ethanol precipitation. HFPS contains 63.56% sulfated polysaccharides, which constitutes by glucose (5.95%), xylose (17.37%), galactose (23.15%), and fucose (53.53%).

### 3.2. Measurement of Enzyme Inhibitory Effects of HFPS

#### 3.2.1. Measurement of Inhibitory Effect on Collagenase from Clostridium Histolyticum

To measure the collagenase inhibitory activity, a weight of 1 mg of azo dye-impregnated collagen was mixed with 800 μL of 0.1 M Tris-HCl (pH 7.0), 100 μL of 200 units/mL collagenase (stock solution), and 100 μL sample and incubated at 43 °C for 1 h under shaking condition. Subsequently, the reaction mixture was centrifuged at 3000 rpm for 10 min and the absorbance of the supernatant was detected at 550 nm in a microplate reader (BioTek Synergy HT, Woburn, MA, USA).

#### 3.2.2. Measurement of Inhibitory Effect on Elastase from Porcine Pancreas

The elastase inhibitory activity was evaluated base on a method reported by Kraunsoe et al. (1996) [41]. In brief, the reaction mixture contained 650 μL of 1.015 mM N-succinyl-Ala-Ala-Ala-p-nitroanilide (dissolved in Tris-HCl, pH 8.0) and 50 μL of sample. The reaction mixture was vortexed and incubated for 10 min at 25 °C. After incubation, a volume of 50 μL of 0.0375 units/mL elastase enzyme solution was added to the reaction mixture and, following vortexing, the reaction mixture was incubated for 10 min at 25 °C in a water bath. The amount of released p-nitroaniline was assessed by measuring absorbance at 410 nm using a microplate reader.

### 3.3. Cell Culture and UVB Irradiation

HDF cells (ATCC^®^ PCS20101™) were purchased from ATCC (American Type Culture Collection, Manassas, VA, USA). HDF cells were cultured in DMEM and F-12 mixed with a ratio of three to one supplemented with 10% heat-inactivated FBS, 100 unit/mL of penicillin and 100 μg/mL of streptomycin. Cells were sub-cultured every 5 days. Cells were incubated at 37 °C under humidified atmosphere containing 5% CO_2_ in an incubator (Sanyo MCO-18AIC CO_2_ Incubator, Moriguchi, Japan). UVB irradiation was carried out using a UVB meter (UV Lamp, VL-6LM, Vilber Lourmat, France), equipped with a fluorescent bulb emitting 280–320 nm wavelength with a peak at 313 nm. HDF cells were irradiated at a dose of 50 mJ/cm^2^ of UVB in 1 × PBS. Cell medium was subsequently replaced with serum free medium and incubated until analysis.

### 3.4. Effect of HFPS on UVB-Irradiated HDF Cells

#### 3.4.1. Cell Viability Assay

Cytotoxicity of HFPS on HDF cells was assessed by a colorimetric MTT assay. Briefly, cells were seeded at a concentration of 5.0 × 10^4^ cells per well in 24-well plates. After 24 h, cells were treated with HFPS (25, 50, and 100 μg/mL) for 48 h, and their viabilities were determined by the method described previously [42,43].

#### 3.4.2. Determination of Intracellular ROS Level and Cell Viability

For intracellular ROS level analysis, HDF cells were treated with HFPS and incubated for 30 min. Subsequently, cells were treated with DCFH-DA (stock, 500 μg/mL) and incubated for 30 min. After incubation, cells were exposed to UVB (50 mJ/cm^2^) and incubated for 1 h at 37 °C. The fluorescence intensity of cells was determined according to the method described previously [44].

To analyze the protective effect of HFPS against UVB-induced cell damage, HDF cells were treated with HFPS and incubated for 2 h at 37 °C. Cells were then exposed to 50 mJ/cm^2^ of UVB and incubated for 48 h. Cell viability was assessed by MTT assay using the protocol described previously [43,45].

#### 3.4.3. Determination of Relative Intracellular Elastase and Collagenase Activities

HDF cells were seeded in 100 mm culture dishes at a density of 2.0 × 10^6^ cells per dish and incubated for 24 h. Cells were pretreated with HFPS and incubated for 2 h. Following incubation, cells were irradiated with UVB. After 48 h incubation, cells were harvested and lysed with 0.1 M Tris-HCl (pH 7.6) buffer containing 1 mM PMSF and 0.1% Triton-X 100, followed by sonication for 5 min on ice. The lysates were centrifuged (4000 rpm, 20 min) at 4 °C. Supernatants were quantified for their protein content and were used as the fibroblastic enzyme solution. The relative elastase and collagenase activities were measured by the method described by Suganuma et al. [46].

#### 3.4.4. Determination of Collagen Synthesis Level and MMPs Expression Levels by Enzyme-Linked Immunosorbent Assay (ELISA)

HDF cells were incubated with HFPS for 2 h, and exposed to UVB (50 mJ/cm^2^). After 48 h incubation, the culture media were collected and used for assessment of MMPs expression levels and PIP level that reflect the level of collagen synthesis. The amounts of PIP and MMPs were measured by commercial ELISA kits, based on the manufacturer’s instructions.

#### 3.4.5. Western Blot Analysis

The effect of HFPS on the expressions of NF-κB, p-c-Jun, and MAPKs were assessed by Western blot analysis performed as described previously [43,47]. In brief, cells were pretreated with HFPS and irradiated with UVB. After 1 h (for MAPKs assay) or 6 h (for NF-κB and p-c-Jun assay) incubation, cells were harvested. Proteins were extracted with the PRO-PREP protein extraction kit (iNtRON Biotechnology, Sungnam, Korea). The protein level of each sample was measured by a BCA™ kit. Total proteins (50 μg) were separated on 10% sodium dodecyl sulfate (SDS)-polyacrylamide gels and transferred to pure nitrocellulose membranes. Membranes were blocked with 5% skim milk for 3 h at room temperature and incubated with primary antibodies overnight at 4 °C. After washing with TBS-T buffer, membranes were incubated with secondary antibodies for 3 h at room temperature. Finally, the protein bands were visualized using an ECL western blotting detection kit and exposed on X-ray films.

### 3.5. Statistical Analysis

The experiments were performed in triplicate. All data are expressed as the mean ± SE. Significant differences between the groups were determined using the unpaired Student’s *t*-test (using Statistical Product and Service Solutions 11.5 statistical software). Values of * *p* < 0.05, ** *p* < 0.01, and ^##^ *p* < 0.01 were considered as significantly different.

## 4. Conclusions

In conclusion, in the present study, the protective effects of HFPS against UVB-induced skin damage *in vitro* in HDF cells were investigated. The results indicate that HFPS significantly protected collagen synthesis and reduced MMPs expression in UVB-irradiated HDF cells by regulating NF-κB, AP-1, and MAPKs signaling pathways. These results suggest that HFPS possess strong UV protective effect and has potential to be used as an ingredient in pharmaceutical and cosmetic industries.

## References

[1] Fisher G.J., Kang S., Varani J., Bata-Csorgo Z., Wan Y., Datta S., Voorhees J.J. (2002). Mechanisms of photoaging and chronological skin aging. Arch. Dermatol..

[2] Longstreth J., De Gruijl F., Kripke M., Abseck S., Arnold F., Slaper H., Velders G., Takizawa Y., Van der Leun J. (1998). Health risks. J. Photochem. Photobiol. B.

[3] Tanaka K., Hasegawa J., Asamitsu K., Okamoto T. (2007). Magnolia ovovata extract and its active component magnolol prevent skin photoaging via inhibition of nuclear factor κB. Eur. J. Pharmacol..

[4] Ryu B., Ahn B.-N., Kang K.-H., Kim Y.-S., Li Y.-X., Kong C.-S., Kim S.-K., Kim D.G. (2015). Dioxinodehydroeckol protects human keratinocyte cells from UVB-induced apoptosis modulated by related genes Bax/Bcl-2 and caspase pathway. J. Photochem. Photobiol. B.

[5] Pathak M.A., Fanselow D.L. (1983). Photobiology of melanin pigmentation: Dose/response of skin to sunlight and its contents. J. Am. Acad. Dermatol..

[6] Wang L., Ryu B., Kim W.-S., Kim G.H., Jeon Y.-J. (2017). Protective effect of gallic acid derivatives from the freshwater green alga *Spirogyra* sp. against ultraviolet B-induced apoptosis through reactive oxygen species clearance in human keratinocytes and zebrafish. Algae.

[7] Katiyar S.K., Bergamo B.M., Vyalil P.K., Elmets C.A. (2001). Green tea polyphenols: DNA photodamage and photoimmunology. J. Photochem. Photobiol. B.

[8] Pallela R., Na-Young Y., Kim S.-K. (2010). Anti-photoaging and photoprotective compounds derived from marine organisms. Mar. Drugs.

[9] Adil M.D., Kaiser P., Satti N.K., Zargar A.M., Vishwakarma R.A., Tasduq S.A. (2010). Effect of *Emblica officinalis* (fruit) against UVB-induced photo-aging in human skin fibroblasts. J. Ethnopharmacol..

[10] Thomas N.V., Kim S.-K. (2010). Metalloproteinase inhibitors: Status and scope from marine organisms. Biochem. Res. Int..

[11] Ko S.-C., Jung W.-K., Lee S.-H., Lee D.H., Jeon Y.-J. (2017). Antihypertensive effect of an enzymatic hydrolysate from Styela clava flesh tissue in type 2 diabetic patients with hypertension. Nutr. Res. Pract..

[12] Ko J.-Y., Kang N., Lee J.-H., Kim J.-S., Kim W.-S., Park S.-J., Kim Y.-T., Jeon Y.-J. (2016). Angiotensin I-converting enzyme inhibitory peptides from an enzymatic hydrolysate of flounder fish (Paralichthys olivaceus) muscle as a potent anti-hypertensive agent. Process Biochem..

[13] Kang M.-C., Kang N., Ko S.-C., Kim Y.-B., Jeon Y.-J. (2016). Anti-obesity effects of seaweeds of Jeju Island on the differentiation of 3T3-L1 preadipocytes and obese mice fed a high-fat diet. Food Chem. Toxicol..

[14] Lee W., Kang N., Kim E.-A., Yang H.-W., Oh J.-Y., Fernando I.P.S., Kim K.-N., Ahn G., Jeon Y.-J. (2017). Radioprotective effects of a polysaccharide purified from Lactobacillus plantarum-fermented Ishige okamurae against oxidative stress caused by gamma ray-irradiation in zebrafish *in vivo* model. J. Funct. Foods.

[15] Lee S.-H., Ko S.-C., Kang M.-C., Lee D.H., Jeon Y.-J. (2016). Octaphlorethol A, a marine algae product, exhibits antidiabetic effects in type 2 diabetic mice by activating AMP-activated protein kinase and upregulating the expression of glucose transporter 4. Food Chem. Toxicol..

[16] Lee W.W., Kim W.S., Ahn G., Kim K.N., Heo S.J., Cho M., Fernando I.P.S., Kang N., Jeon Y.-J. (2016). Separation of glycine-rich proteins from sea hare eggs and their anti-cancer activity against U937 leukemia cell line. Exeli J..

[17] Oh J.-Y., Fernando I.S., Jeon Y.-J. (2016). Potential applications of radioprotective phytochemicals from marine algae. Algae.

[18] Sanjeewa K.K.A., Fernando I.P.S., Samarakoon K.W., Lakmal H.H.C., Kim E.-A., Kwon O.N., Dilshara M.G., Lee J.-B., Jeon Y.-J. (2016). Anti-inflammatory and anti-cancer activities of sterol rich fraction of cultured marine microalga Nannochloropsis oculata. Algae.

[19] Kim H.-H., Kim H.-S., Ko J.-Y., Kim C.-Y., Lee J.-H., Jeon Y.-J. (2016). A single-step isolation of useful antioxidant compounds from Ishige okamurae by using centrifugal partition chromatography. Fish Aquat. Sci..

[20] Kang N., Kim S.-Y., Rho S., Ko J.-Y., Jeon Y.-J. (2017). Anti-fatigue activity of a mixture of seahorse (Hippocampus abdominalis) hydrolysate and red ginseng. Fish. Aquatic. Sci..

[21] Park S.-Y., Hwang E., Shin Y.-K., Lee D.-G., Yang J.-E., Park J.-H., Yi T.-H. (2017). Immunostimulatory effect of enzyme-modified *Hizikia fusiformein* a mouse model *in vitro* and ex vivo. Mar. Biotechnol..

[22] Baek J., Lim S.-Y. (2017). Effect of *Hizikia fusiformis* extracts on reactive oxygen species mediated oxidative damage. Int. J. Adv. Res. Biol. Sci..

[23] Imbs T.I., Ermakova S.P., Malyarenko O.S., Isakov V.V., Zvyagintseva T.N. (2016). Structural elucidation of polysaccharide fractions from the brown alga *Coccophora langsdorfii* and *in vitro* investigation of their anticancer activity. Carbohyd. Polym..

[24] Oh J.-H., Kim J., Lee Y. (2016). Anti-inflammatory and anti-diabetic effects of brown seaweeds in high-fat diet-induced obese mice. Nutr. Res. Pract..

[25] Wang L., Oh J.Y., Kim H.S., Lee W., Cui Y., Lee H.G., Kim Y.-T., Ko J.Y., Jeon Y.-J. (2018). Protective effect of polysaccharides from Celluclast-assisted extract of *Hizikia fusiforme* against hydrogen peroxide-induced oxidative stress *in vitro* in Vero cells and *in vivo* in zebrafish. Int. J. Biol. Macromol..

[26] Heo S.-J., Ko S.-C., Cha S.-H., Kang D.-H., Park H.-S., Choi Y.-U., Kim D., Jung W.-K., Jeon Y.-J. (2009). Effect of phlorotannins isolated from Ecklonia cava on melanogenesis and their protective effect against photo-oxidative stress induced by UV-B radiation. Toxicol. In Vitro.

[27] Ko S.-C., Cha S.-H., Heo S.-J., Lee S.-H., Kang S.-M., Jeon Y.-J. (2011). Protective effect of Ecklonia cava on UVB-induced oxidative stress: In vitro and *in vivo* zebrafish model. J. Appl. Physiol..

[28] Zeng Q., Zhou F., Lei L., Chen J., Lu J., Zhou J., Cao K., Gao L., Xia F., Ding S. (2017). *Ganoderma lucidum* polysaccharides protect fibroblasts against UVB-induced photoaging. Mol. Med. Rep..

[29] Plastow S.R., Lovell C.R., Young A.R. (1987). UVB-Induced collagen changes in the skin of the hairless albino mouse. J. Invest. Dermatol..

[30] Imokawa G. (2009). Mechanism of UVB-Induced wrinkling of the skin: Paracrine cytokine linkage between keratinocytes and fibroblasts leading to the stimulation of elastase. J. Invest. Dermatol. Symp. Proc..

[31] Li Y.H., Wu Y., Wei H.C., Xu Y.Y., Jia L.L., Chen J., Yang X.S., Dong G.H., Gao X.H., Chen H.D. (2009). Protective effects of green tea extracts on photoaging and photommunosuppression. Skin Res. Technol..

[32] Nikkari S.T., O’Brien K.D., Ferguson M., Hatsukami T., Welgus H.G., Alpers C.E., Clowes A.W. (1995). Interstitial collagenase (MMP-1) expression in human carotid atherosclerosis. Circulation.

[33] Zhang M., Hwang E., Lin P., Gao W., Ngo H.T., Yi T.-H. (2018). Prunella vulgaris L. exerts a protective effect against extrinsic aging through NF-κB, MAPKs, AP-1, and TGF-β/Smad signaling pathways in UVB-Aged normal human dermal fibroblasts. Rejuvenation Res..

[34] Hwang B.-M., Noh E.-M., Kim J.-S., Kim J.-M., Hwang J.-K., Kim H.-K., Kang J.-S., Kim D.-S., Chae H.-J., You Y.-O. (2013). Decursin inhibits UVB-induced MMP expression in human dermal fibroblasts via regulation of nuclear factor-κB. Int. J. Mol. Med..

[35] Cooper S., Bowden G. (2007). Ultraviolet B regulation of transcription factor families: Roles of nuclear factor-kappa B (NF-κB) and activator protein-1 (AP-1) in UVB-induced skin carcinogenesis. Curr. Cancer Drug Targets.

[36] Bell S., Degitz K., Quirling M., Jilg N., Page S., Brand K. (2003). Involvement of NF-κB signalling in skin physiology and disease. Cell Signal..

[37] Zhang W., Liu H.T. (2002). MAPK signal pathways in the regulation of cell proliferation in mammalian cells. Cell Res..

[38] Rodríguez-Berriguete G., Fraile B., Martínez-Onsurbe P., Olmedilla G., Paniagua R., Royuela M. (2012). MAP kinases and prostate cancer. J. Signal. Ttransduct..

[39] Cargnello M., Roux P.P. (2011). Activation and function of the MAPKs and their substrates, the MAPK-activated protein kinases. Microbiol. Mol. Biol. Rev..

[40] Pittayapruek P., Meephansan J., Prapapan O., Komine M., Ohtsuki M. (2016). Role of matrix metalloproteinases in photoaging and photocarcinogenesis. Int. J. Mol. Sci..

[41] Kraunsoe J.A., Claridge T.D., Lowe G. (1996). Inhibition of human leukocyte and porcine pancreatic elastase by homologues of bovine pancreatic trypsin inhibitor. Biochemistry.

[42] Kang M.-C., Kim S.-Y., Kim E.-A., Lee J.-H., Kim Y.-S., Yu S.-K., Chae J.B., Choe I.-H., Cho J.H., Jeon Y.-J. (2015). Antioxidant activity of polysaccharide purified from *Acanthopanax koreanum* Nakai stems *in vitro* and *in vivo* zebrafish model. Carbohyd. Polym..

[43] Wang L., Fernando I.S., Kim E.-A., Jeon Y.-J. (2016). Soft corals collected from Jeju Island; a potential source of anti-inflammatory phytochemicals. J. Chitin Chitosan.

[44] Heo S.-J., Jeon Y.-J. (2009). Protective effect of fucoxanthin isolated from Sargassum siliquastrum on UV-B induced cell damage. J. Photochem. Photobiol. B.

[45] Wang L., Jo M.-J., Katagiri R., Harata K., Ohta M., Ogawa A., Kamegai M., Ishida Y., Tanoue S., Kimura S. (2018). Antioxidant effects of citrus pomace extracts processed by super-heated steam. LWT-Food Sci. Technol..

[46] Suganuma K., Nakajima H., Ohtsuki M., Imokawa G. (2010). Astaxanthin attenuates the UVA-induced up-regulation of matrix-metalloproteinase-1 and skin fibroblast elastase in human dermal fibroblasts. J. Dermatol. Sci..

[47] Lee S.-H., Han J.-S., Heo S.-J., Hwang J.-Y., Jeon Y.-J. (2010). Protective effects of dieckol isolated from *Ecklonia cava* against high glucose-induced oxidative stress in human umbilical vein endothelial cells. Toxicol. In Vitro.

